# A Controlled in Silico Benchmark for GNN Prediction of Tissue Dynamics

**DOI:** 10.21203/rs.3.rs-8575848/v2

**Published:** 2026-07-13

**Authors:** Matej Krajnc, Troy Comi, Siqi Miao, Adnan Hafeez, Hadar Serviansky, Pan Li, Tomer Stern

**Affiliations:** 1 Department of Theoretical Physics, Jožef Stefan Institute, Ljubljana, Slovenia; 2 The Lewis-Sigler Institute for Integrative Genomics, Princeton University, Princeton, New Jersey, USA; 3 School of Electrical and Computer Engineering, Georgia Institute of Technology, Atlanta, Georgia, USA; 4 Advanced Research Computing, Information and Technology Services, University of Michigan, Ann Arbor, Michigan, USA; 5 Department of Computer Science and Applied Mathematics, Weizmann Institute of Science, Rehovot, Israel; 6 Department of Biologic and Materials Sciences, University of Michigan, Ann Arbor, Michigan, USA

## Abstract

Graph Neural Networks (GNNs) are promising tools for predicting tissue dynamics, but choosing the right architecture remains difficult because experimental datasets are limited, noisy, and system-specific. We introduce a controlled in silico benchmark for comparing GNN architectures on a vertex-model task: predicting relaxed cell-cell interface lengths after a cell neighbor exchange. Because simulated data provide known ground truth, we can vary tissue geometry, mechanics, perturbation complexity, dataset size, and input features independently. Provably Powerful Graph Networks (PPGN) and Principal Neighborhood Aggregation (PNA) were most sample-efficient when pre-event edge lengths were provided, whereas performance dropped sharply with topology alone. We also revealed a predict-or-copy strategy, whereby predictions far from the exchange copied pre-event lengths instead of predicting long-range changes. Prediction was harder in disordered tissues, suggesting hexagonality as a simple indicator of difficulty. These results provide a reproducible testbed for diagnosing feature dependence, copying behavior, and geometric consistency in tissue-remodeling prediction.

## INTRODUCTION

Understanding how cells organize into complex tissues is a primary goal in developmental biology, with far-reaching implications for clinical applications in cancer^1^, wound healing^2^, and beyond^3^. Tissue dynamics is challenging to unravel because cell morphology, short- and long-range mechanical forces, and molecular signals interact simultaneously across many spatial and temporal scales^4–6^. Each of these factors has independently posed experimental and computational challenges for decades. A core difficulty arises from the dynamic connectivity among cells: neighbor relationships define the network through which local mechanical and biochemical changes propagate across the tissue. While much progress has been made in several well-studied model systems^5,7–9^, advancing our understanding of tissue dynamics remains highly challenging, and existing models typically capture only a fraction of the elements involved^10,11^. This motivates automated, systematic methods that can learn from graph-structured tissue measurements while still being evaluated in controlled settings where the relevant sources of difficulty can be isolated. Here we focus on developing epithelia, where tissue remodeling during morphogenesis depends on coordinated changes in cell-cell connectivity, junctional geometry, and tissue-scale mechanical context.

A prominent example of collective cell behavior is cell intercalation, also known as a T1 transition, where a shared edge between two adjacent cells shrinks and disappears, and a new edge forms between their neighboring cells, effectively rearranging the cell connectivity. These rearrangements are a hallmark of embryonic morphogenesis, underpinning key developmental processes such as tissue elongation and axis formation across species^12^, as well as wound healing^13^. For example, during early *Drosophila* germband extension, polarized T1 transitions enable the embryo to elongate without cell proliferation^14,15^, while in epithelial wound healing, similar events restore tissue integrity by allowing cells to flow past one another via intercalations^13^. These intercalations effectively fluidize the tissue, transforming it from a solid-like to a more pliable state^13,16^. In these developmental contexts, accurate full-state prediction benefits from incorporating nonlocal context spanning multiple cell diameters, consistent with the long-range mechanical and biochemical coordination observed in vivo. These examples motivate treating T1 transitions as localized topological perturbations embedded in a mechanically coupled tissue network. A T1 is not only a local edge replacement: it changes the neighborhood relationships among cells and can alter the geometry of surrounding interfaces through force balance, junctional tension, adhesion, and tissue-scale loading^17–21^. This makes T1 transitions a useful test case for graph-based prediction, because the input and output are naturally defined on a cell-contact network and the response can be evaluated as a spatially distributed change in edge lengths.

Mechanistic models are central for interpreting tissue dynamics because they connect motion to explicit biophysical quantities such as junctional tension, adhesion, cortical contractility, and external loading^20–25^. Graph neural networks (GNN) offer a complementary route: rather than specifying the full dynamical law, they learn graph-structured input-output mappings from examples^26,27^. This is attractive for biological tissues because many measurable quantities—cell and junction geometry, local motion, inferred mechanical variables, gene or protein expression, and tissue-level context—could in principle be combined in a single predictive model. However, this flexibility creates a benchmarking problem: model performance can depend strongly on architecture, feature availability, tissue structure, and the spatial range over which information must propagate.

GNNs operate directly on graph-structured data, making them well matched to tissues in which cells form nodes and cell-cell interfaces form edges^26,28^. In message-passing neural networks (MPNN), node or edge representations are iteratively updated by aggregating information from neighboring cells or interfaces, so deeper models can in principle integrate progressively broader tissue context^29–32^. Higher-order architectures such as Provably Powerful Graph Networks (PPGN) instead operate on pairwise node-node representations, increasing expressive power at greater computational cost^33^. These architectural differences matter for tissue prediction because local remodeling responses may depend not only on immediate neighbors, but also on tissue disorder, geometry, and longer-range mechanical context.

Recent studies have shown that GNNs can extract predictive information from live tissue data, including cell-behavior classification in mouse epidermis^34^ and multicellular event forecasting in whole-embryo *Drosophila* datasets^9,35^. These studies demonstrate the practical value of graph-based learning for biological tissues: geometry, local motion, molecular markers, and tissue context can be represented together without requiring a complete mechanistic equation for their interactions. However, they also leave open a more general benchmarking question. Because each experimental dataset has its own tissue geometry, feature set, noise structure, annotation process, and biological regime, performance on one dataset does not by itself reveal which architecture, features, or spatial scales are responsible for successful prediction. This is especially limiting for full-state geometric prediction, where the goal is not only to classify a local event but to reconstruct the tissue-wide response to a specified perturbation.

Addressing this gap requires a benchmark in which the prediction target, perturbation, tissue structure, feature availability, and training-set size are all known by construction. A controlled simulated tissue system provides such a setting. It does not replace experimental validation, but it can isolate why a model succeeds or fails in ways that are difficult to determine from live-imaging datasets alone. This is especially useful for the task considered here, where model performance may depend not only on local event cues but also on tissue disorder, mechanical regime, and the spatial extent of the relaxation response.

In this study, we introduce a deliberately controlled in silico benchmark for evaluating how GNN architectures learn tissue-wide geometric responses to local topological perturbations. Each sample is a two-dimensional confluent tissue generated by a vertex model. A T1 transition is imposed on a specified cell-cell interface, the tissue is relaxed to a new mechanical equilibrium, and the supervised target is the relaxed post-event length of every interface. The resulting task is a full-state regression problem: given a graph-structured representation of the tissue and perturbation, predict the continuous edge-length field after relaxation. In this benchmark, we compare four MPNN architectures—GraphSAGE^32^, Graph Attention Network (GAT)^31^, Graph Isomorphism Network (GIN)^30^, and Principal Neighborhood Aggregation (PNA)^29^—with the higher-order Provably Powerful Graph Networks (PPGN)^33^ architecture.

This formulation deliberately separates conditional-response prediction from event discovery. The model is not asked to determine which interface will remodel, nor to predict the timing of a T1 event in a living embryo. Instead, the benchmark asks how accurately different architectures can infer the downstream geometric response once a local perturbation has been specified. Because the data are generated in simulation, we can vary tissue disorder, system size, area elasticity, imposed shear, perturbation number, distance from the perturbation, training-set size, and feature availability while retaining known post-perturbation ground truth. The benchmark therefore sacrifices biological completeness in exchange for a controlled setting in which architecture, feature, and tissue-regime effects can be isolated.

In the edge-featured version of the benchmark, the specified T1 interface is represented by a binary edge feature. This feature should be interpreted as a controlled conditioning variable rather than an oracle-like label for natural remodeling events. Biologically, analogous conditioning arises when the location of a remodeling cue or perturbation is known in advance or measured post hoc, such as planar-polarized actomyosin and adhesion patterns during *Drosophila* germband extension^18,19^, molecularly patterned contractility during junction remodeling^20,21^, graded signaling effects on tissue mechanics^6,36^, or experimentally imposed optogenetic contractility^37^.

## RESULTS

Our objective is to evaluate how effectively different GNN architectures capture the dynamics of evolving two-dimensional (2D) tissues and predict their future states. We focus on prediction accuracy as a function of multiple architectural, dataset-level, and tissue-level factors: (i) training-set size, (ii) the ability to integrate nonlocal information, quantified by the effective receptive field, (iii) the importance of explicitly providing edge-length features that could, in principle, be inferred from the data, (iv) mechanical regime, varied through the area-elasticity coefficient $k_{A}$, (v) global tissue-scale shear, (vi) tissue size, (vii) tissue disorder, quantified by hexagonality, the fraction of cells with six neighbors, (viii) perturbation complexity, comparing single- and two-T1 events, and (ix) the ability to realize predicted edge lengths as a consistent two-dimensional tissue embedding.

**Figure 1.**
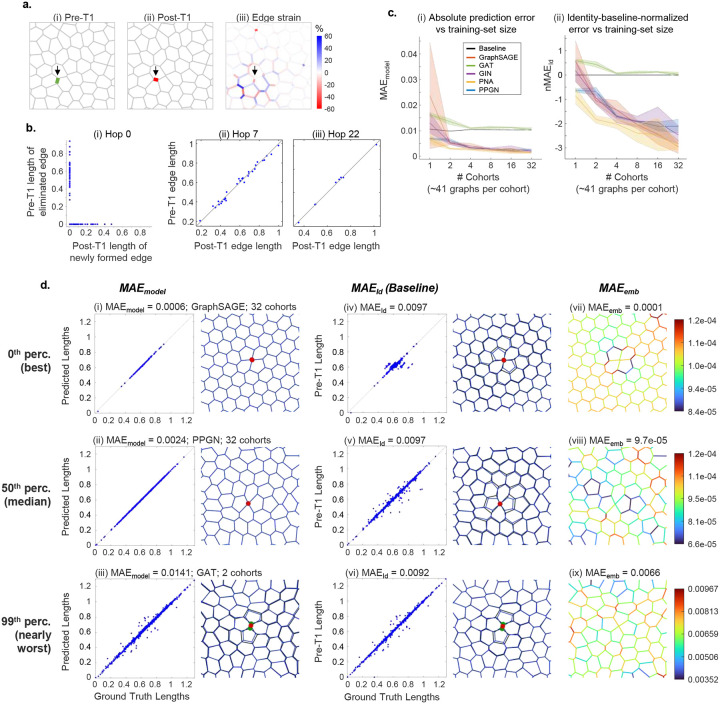
GNNs learn a controlled post-T1 edge-length prediction task from limited simulated tissue data. **a.** Example of the benchmark T1 perturbation. (i) pre-T1 configuration with the selected interface in green; (ii) relaxed post-T1 configuration with the newly formed interface in red; and (iii) edge-strain map showing percent length change after relaxation, with red indicating shortening and blue elongation. **b.** Edge-length changes decay with edge-hop distance from the T1 interface. (i) Hop 0 contains two point clouds because the T1 edge is replaced: eliminated interfaces have nonzero pre-T1 length but zero post-T1 length, giving the vertical cloud at x = 0, whereas newly formed interfaces have zero pre-T1 length but nonzero post-T1 length, giving the horizontal cloud at y = 0. Because each graph has only one such pair, points are pooled from 50 randomly sampled test graphs. (ii–iii) Hops 7 and 22 compare matched interfaces from the same graph. Example tissues show the test graph with the largest pre- to post-T1 deviation at each hop. **c.** Model performance versus training-set size. (i) Mean graph-level absolute error for five GNN architectures trained on 1–32 cohorts, computed by first averaging edge-level absolute errors within each graph and then averaging graph-level $MAE_{\text{model}}$ values across test graphs and seeds. The identity baseline predicts post-T1 edge lengths as unchanged from pre-T1. (ii) Identity-baseline-normalized error, computed as graph-level ${log}_{2}$ ratios of $MAE_{\text{model}}(G)$ to $MAE_{Id}\left(G_{k}\right)$, followed by averaging across test graphs and seeds; values below zero indicate improvement over baseline. Curves show mean ± s.d. across n=5 random seeds. **d.** Representative best, median, and 99^th^-percentile test examples ranked by graph-level prediction error. For each graph, subpanels show, from left to right: predicted versus ground-truth post-T1 edge lengths; the corresponding embedded prediction overlaid on the ground-truth tissue; pre-T1 versus ground-truth post-T1 edge lengths for the identity baseline; the corresponding pre-T1 tissue (baseline) overlaid on the ground truth; and edge-wise embedding residuals for the predicted tissue. In the embedding, predicted or pre-T1 geometries are blue, ground-truth post-T1 geometries are black, eliminated T1 interfaces are green, and newly formed T1 interfaces are red.

To isolate these factors, we employ controlled 2D vertex-model simulations. In the main benchmark, each simulation follows a three-step protocol (Fig. 1a): the tissue is first relaxed to equilibrium, a randomly selected T1 event (edge exchange) is imposed, and the system is relaxed again to its new minimum-energy state. This controlled perturbation allows us to quantify how a local rearrangement propagates through the tissue: immediately adjacent edges exhibit large changes, while those farther away show progressively smaller deviations (Fig. 1b). The spread of these fluctuations with hop distance defines the spatial structure of the prediction task and allows us to evaluate how prediction accuracy changes with distance from the perturbation. We then extend this framework to ask how prediction accuracy changes with tissue disorder, explicitly provided edge-length information, mechanical parameters, global shear, tissue size, perturbation complexity, and the 2D realizability of predicted edge lengths. To improve statistical reliability, each training condition was repeated with n=5 independent random seeds. Unless otherwise noted, results are reported as mean ± standard deviation (s.d.) across these runs. All simulations and computational procedures—including vertex-model simulation, dataset generation and splitting, GNN architectures and implementations, hyperparameter optimization, and 2D embedding post-processing—are detailed in the Methods section.

### GNNs learn post-T1 edge-length prediction from small training sets, with architecture rankings depending on data regime:

We first compared the accuracy of the five GNN architectures as a function of training set size ranging from one to 32 simulation cohorts, corresponding to approximately 41 to 1,298 training graphs (Fig. 1c(i)). This analysis asks how well the post-T1 edge-length prediction task can be learned from pre-T1 edge-length and tissue topology, and whether different architectures differ in sample efficiency. For this edge-featured input regime, hyperparameters for each architecture were optimized on the 16-cohort reference dataset and then held fixed across the training-set-size comparisons (Methods; Supplementary Table S1). As the performance metric, we used the mean absolute error (MAE) per graph $G=\left(V_{G},E_{G}\right)$, computed as the L1 difference between predicted (${\overset{ˆ}{l}}_{e}$) and true ($l_{e}^{\text{post}}$) post-T1 edge lengths averaged over all edges in the graph:

(1) $${MAE}_{\text{model}}(G)=\frac{1}{\left|E_{G}\right|}\sum_{e\in E_{G}}\left|{\overset{ˆ}{l}}_{e}-l_{e}^{\text{post}}\right|.$$

To contextualize prediction quality, we normalized $MAE_{\text{model}}$ against an identity baseline that estimates each post-T1 edge length as its corresponding pre-T1 length. This baseline represents the error expected from a model that does not learn a post-T1 update rule and instead copies the input edge length as its best estimate. We compute a single identity denominator for each training-set-size condition as the average identity MAE over all test graphs $G_{k}$ for that condition:

(2) $${MAE}_{Id}\left(G_{k}\right)=\frac{1}{\left|G_{k}\right|}\sum_{G\in G_{k}}\frac{1}{\left|E_{G}\right|}\sum_{e\in E_{G}}\left|l_{e}^{pre}-l_{e}^{\text{post}}\right|.$$

where $G_{k}$ is the set of all test graphs from the subset of size k. This baseline requires no training and quantifies the inherent difficulty of predicting Δ-lengths. For each test graph G, model, and random seed, the graph-level normalized-$MAE_{Id}(nMAE_{Id})$ is given by:

(3) $$nMAE_{Id,S}\left(G,G_{k}\right)={log}_{2}\left(\frac{MAE_{\text{model},s}(G)}{MAE_{Id}\left(G_{k}\right)}\right).$$

The plotted $nMAE_{Id}$ value is obtained by averaging these graph-level log-ratios over all test graphs in $G_{k}$, and then across the n=5 random seeds:

(4) $$nMAE_{Id}\left(G_{k}\right)=\frac{1}{5}\sum_{s=1}^{5}\frac{1}{\left|G_{k}\right|}\sum_{G\in G_{k}}nMAE_{Id,S}\left(G,G_{k}\right).$$

We use a base-2 logarithm directly in the graph-level definition to obtain a symmetric, dimensionless measure in which zero indicates parity with the baseline, negative values indicate improvement, and positive values indicate worse performance (Fig. 1c(ii)). Because averaging is performed after taking the graph-level logarithm, the plotted $nMAE_{Id}$ is equivalent to the ${log}_{2}$ of a geometric mean of graph-level model-to-identity MAE ratios. It is therefore not, in general, equal to the ${log}_{2}$ ratio of the arithmetic mean $MAE_{\text{model}}$ to the arithmetic mean $MAE_{Id}$. To calibrate these scales, Fig. 1d shows representative scatter plots and 2D embeddings from the 99^th^-percentile (nearly worst), median, and best test graphs; the visual differences mirror the numerical hierarchy.

Across training-set sizes, prediction accuracy generally improved with additional data, indicating that the task is learnable and that the models capture a nontrivial mapping from pre-T1 tissue geometry to relaxed post-T1 edge lengths. This improvement was already apparent in the low-data regime: PNA and PPGN outperformed the identity baseline with only 41 training graphs (1 cohort), while GraphSAGE and GIN required 81 training graphs to cross the baseline, and GAT remained close to the identity baseline. For PNA, this low-data performance corresponded to a mean ± s.d. $MAE_{\text{model}}$ of 0.0065 ± 0.0018 across n=5 random seeds, compared with an average identity-baseline $MAE_{Id}$ of 0.0106. The corresponding plotted value was $nMAE_{Id}=-0.8489\pm 0.3293$, computed by first applying the seed-specific graph-level definition:

(5) $$nMAE_{Id,S}\left(G,G_{k}\right)={log}_{2}\left(\frac{MAE_{\text{model},s}(G)}{MAE_{Id}\left(G_{k}\right)}\right),$$

then averaging these graph-level log-ratios across test graphs and seeds.

Across models, performance continued to improve as additional cohorts were added, reaching $nMAE_{Id}=-2.9546\pm 0.2669$ for GraphSAGE, −2.9462 ± 0.1357 for PNA, −2.4687 ± 0.3723 for GIN, and −2.1037 ± 0.2770 for PPGN at 1,298 training graphs. In contrast, GAT improved only modestly, from 0.5954 ± 0.0666 with 41 training graphs to 0.0345 ± 0.0799 with 1,298 training graphs, and never robustly outperformed the identity baseline. Thus, the main architectural difference in the low-data regime is sample efficiency: PNA and PPGN learn useful post-T1 edge-length predictions from very limited data, whereas GraphSAGE and GIN require additional examples before doing so; with larger datasets, GraphSAGE and PNA achieve comparable best performance.

### Effective receptive field scales with dataset size:

To understand how well different GNNs integrate long-range information in biological tissue settings, we analyzed prediction error as a function of topological distance from the T1. This is a particularly relevant test case for modeling biological tissues, as local events can be influenced by tissue-scale mechanical coupling and long-range morphogenetic information flow^36,38^. Because our benchmark contains an isolated T1 perturbation in a 256-cell tissue, it provides a controlled setting for measuring how prediction accuracy changes with distance from the event, over edge-hop distances ranging from 0, the remodeled interface itself, to 22–23 hops.

**Figure 2.**
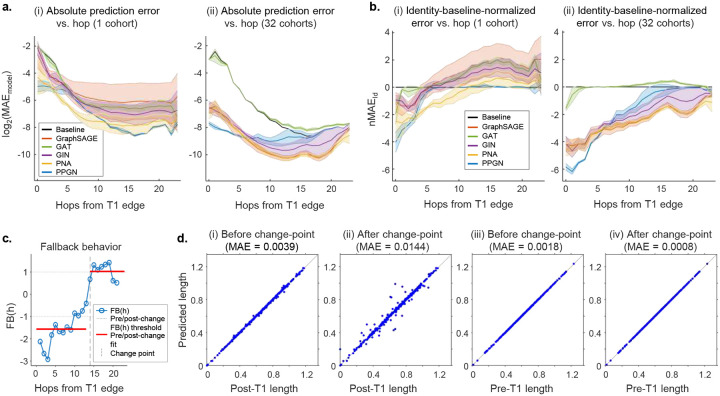
Spatial error profiles reveal hop-dependent prediction accuracy and fallback to the pre-T1 edge lengths. **a.** Log-transformed prediction error versus edge-hop distance from the T1 interface. Edge-hop distance is measured on the line graph of the tissue-contact network, so each hop moves between adjacent cell-cell interfaces. For each hop distance h, edge-level absolute errors were first averaged within each graph to obtain $MAE_{\text{model},s}(h;G)$. The plotted raw-error value is ${log}_{2}\left(MAE_{\text{model}}\left(h;G_{k}\right)\right)$, computed by applying ${log}_{2}$ after averaging graph-level $MAE_{\text{model},\text{s}}(h;G)$ values across test graphs within each seed, followed by averaging across n=5 random seeds. The identity baseline predicts post-T1 edge lengths as unchanged from pre-T1. Curves show mean ± s.d. across n=5 random seeds. **b.** Identity-baseline-normalized prediction error versus edge-hop distance for the same comparisons in a. For each hop distance $h,nMAE_{Id}$ was computed as graph-level ${log}_{2}$ ratios of $MAE_{\text{model},s}(h;G)$ to the hop-specific identity denominator $MAE_{Id}\left(h;G_{k}\right)$, followed by averaging across test graphs and seeds; values below zero indicate improvement over predicting no edge-length change. Normalized performance is strongest near the T1 and becomes less favorable at larger distances, where pre- and post-T1 lengths differ weakly. **c.** Example PPGN fallback detection using the fallback score FB(h), which compares prediction error relative to post-T1 ground truth versus pre-T1 input lengths at each hop. Negative values indicate predictions closer to the post-T1 ground truth; positive values indicate predictions closer to pre-T1 lengths. Fallback was defined by a single change point separating an early region with mean FB(h)≤-1 from a later region with mean FB(h)≥+1. The example shown is the weakest PPGN fallback case among test-graph/seed predictions satisfying this criterion and therefore represents a conservative lower-bound example of the phenomenon. The dashed line marks the detected change point, and red horizontal lines show mean FB(h) before and after it. Hop 0 is excluded because the T1 interface is replaced. **d.** Scatter-plot decomposition of the fallback example in c. The same PPGN prediction is split into edges before (i, iii) and after (ii, iv) the detected change point. Predictions are compared either with post-T1 ground truth (i–ii) or with pre-T1 input lengths (iii–iv). After the change point, predictions are closer to pre-T1 lengths than to post-T1 ground truth, demonstrating fallback toward the input geometry.

To investigate spatial prediction dynamics, we examined the smallest and largest training sets (Fig. 2a; intermediate sizes shown in Supplementary Figure S1). For the raw-error profiles, we plotted ${log}_{2}\left(MAE_{\text{model}}\right)$ after first averaging edge-level absolute errors within each graph and then averaging graph-level $MAE_{\text{model}}$ values across test graphs. For the identity-normalized profiles, we plotted $nMAE_{Id}$, computed as graph-level ${log}_{2}$ ratios to the hop-specific identity denominator and then averaged across test graphs and seeds. Unless otherwise stated, hop-wise curves use the error metrics and T1-relative hop distances defined in Methods. Across all models, absolute error (i.e., ${\text{log}}_{2}\left(MAE_{\text{model}}\right)$) peaked at hops 0 or 1, and dropped sharply over the first several hops. At larger distances, the curves reached a lower-error distal regime, although they were not strictly monotonic and sometimes showed a shallow increase at the farthest hops. This drop, as demonstrated also by the baseline curve, reflects the spatial extent of the mechanical perturbation: edges farther from the T1 remain largely unchanged, making them easier to predict in absolute terms. In contrast, when considering normalized error relative to the identity baseline (i.e., $nMAE_{Id}$; Fig. 2b), we observed the inverse trend: predictions were most accurate near the T1—where the deformation is strongest—and became progressively less effective with distance. This likely reflects both the reduced informativeness of distant edges together with the minimal differences between pre- and post-T1 lengths at those locations, making improvement over the identity baseline increasingly difficult.

To describe the spatial extent over which models outperform the identity baseline, we refer to the effective receptive field (ERF) as the range of hop distances for which $nMAE_{Id}$ remains below zero. At the smallest dataset size (41 training graphs), this below-baseline region was limited: the mean $nMAE_{Id}$ curve first reached or exceeded zero at approximately hop 5 for GAT, hop 6 for GIN and GraphSAGE, and hop 12 for PNA and PPGN. Increasing the training set expanded this effective range. By 650 training graphs, the mean curves for all models except GAT remained below zero across the full measured distance range; GAT still reached the baseline at distal hops even with 1,298 training graphs. Thus, additional data improved not only prediction accuracy near the T1 event, but also the distance over which models could exploit nonlocal tissue information. This distal loss of below-baseline performance at small training-set size is unlikely to reflect a hard architectural receptive-field limit. All MPNN models were trained with 16 layers. In the edge-featured tissues, the full measured range of the interface-hop analysis is approximately 21–23 edge hops from the T1, but those distal interfaces are only approximately 9–11 cell-node hops from the T1 edge endpoints, corresponding to roughly 2.2 interface hops per cell-graph hop. Thus, 16 message-passing layers are sufficient to span the full analyzed tissue range, and the zero crossing at small dataset size more likely reflects limited learned use of available nonlocal context rather than an inability of the network to access those regions.

### A predict-or-copy switch:

While analyzing the $nMAE_{Id}$ profiles (Fig. 2b), we noticed that multiple models showed an abrupt change in accuracy trend as a function of hop distance. This was most noticeable with PPGN, which at the smallest training set size, once reaching the ERF hop, rather than continuing to worsen, its $nMAE_{Id}$ curve merged with the baseline, suggesting that the model begins to output the pre-T1 edge length instead of a new prediction. A similar behavior was evident for PPGN in all dataset sizes, as well as for other models, particularly in intermediate dataset sizes (see Supplementary Fig. S1b).

To quantify this behavior, we defined a fallback score:

(6) $$FB(h)={\text{log}}_{2}\left(\frac{MAE_{\text{post}}(h)}{MAE_{pre}(h)}\right),$$

where $MAE_{\text{post}}(h)$ and $MAE_{\text{pre}}(h)$ are mean absolute errors to post- and pre-T1 lengths, respectively, over all edges $E_{h}$ at hop h. Values FB(h)<0 indicate predictions closer to the ground truth, and FB(h)>0 indicate closer alignment with the pre-T1 lengths. We identified the transition hop $h^{*}$ as a single change point in the FB(h) profile (MATLAB *findchangepts*, statistic = “mean”). A graph was classified as showing fallback when $FB\left(h<h^{*}\right)\leq -1$, and $FB\left(h\geq h^{*}\right)\geq 1$, that is, average prediction was at least twice as close to the post-T1 (ground truth) before hop $h^{*}$ and twice as close to the pre-T1 (baseline) from it. Figures 2c and 2d show the test graph least strongly classified as fallback—i.e., the one closest to the decision threshold—serving as a conservative lower bound on the strength of the phenomenon. Applying this criterion across dataset sizes showed that fallback was strongest and most persistent in PPGN, where it occurred in 70%, 74%, 96%, 94.5%, 86.3%, and 94.0% of test graph–seed predictions from the smallest to largest dataset sizes, respectively. Other models also showed fallback, particularly at intermediate dataset sizes: across the 2- to 32-cohort conditions, GIN reached fallback rates of 24%, 52%, 64%, 34.9%, and 33.2% of test graph × 5 seed predictions; PNA and GraphSAGE showed weaker fallback, peaking at 28.5% and 24.0%, respectively. GAT rarely satisfied the switch-like fallback criterion, because its FB profiles were often already positive from the first nonzero hops rather than showing a transition from post-T1-like predictions near the event to pre-T1-like predictions at distal hops.

To test whether distal fallback was true input copying, we asked a simple counterfactual question: *if we change only the supplied pre-T1 edge length, does the model’s prediction change accordingly?* We used the 16-cohort edge-featured reference data and modified only test-set inputs. For interfaces at edge-hop distance 14 or greater from the newly formed T1 interface, we randomly added either +0.05 or −0.05 to the supplied pre-T1 length, while leaving topology, data splits, model weights, and true post-T1 targets unchanged. Without retraining the models, we then predicted post-T1 edge lengths on these modified inputs. For each perturbed interface, we compared the prediction shift (prediction from modified input − prediction from original input) with the injected input shift (modified pre-T1 length − original pre-T1 length). Copying was quantified by the zero-intercept slope of the prediction shift versus the injected input shift, where a slope of 1 indicates perfect copying, together with the mean residual difference between the two shifts. In all models, prediction shifts closely matched the injected input shifts, and the perturbed-input predictions moved away from the original unperturbed ground-truth post-T1 edge lengths (Table 1). This pattern confirms that distal fallback reflects near-literal copying of the supplied pre-T1 edge length.

We next asked whether fallback depends on architecture-specific shortcut paths that allow models to reuse the supplied pre-T1 edge length. To this end, for the MPNNs, we removed raw edge attributes from the final edge-regression head while leaving the message-passing backbone otherwise intact; edge features could still be used during message passing where normally used, and Jumping Knowledge concatenation was retained. Thus, this ablation specifically removed direct final-head access to the pre-T1 interface length. For PPGN, which does not use the same MPNN-style final edge head, we instead disabled only the first raw-input skip connection while leaving the raw inputs and the later inter-block skip connections intact; these later skips concatenate hidden representations between blocks, not the original raw input features. The MPNN ablations strongly disrupted fallback: distal normalized-error curves no longer approached the identity baseline and instead became worse than identity (Supplementary Fig. S3). In contrast, PPGN fallback was only mildly affected. The original PPGN model showed fallback in 341/395 graph-seed instances (86.3%; mean transition hop 13.43, median 14), whereas the perturbed model showed fallback in 257/316 instances (81.3%; mean transition hop 12.34, median 12). Thus, fallback in MPNNs appears to depend strongly on direct final-head access to pre-T1 length, whereas PPGN can preserve interface-specific length information internally through its pairwise hidden representation. Notably, under these ablated settings, PPGN retained substantially stronger hop-resolved performance than the MPNNs, suggesting that its pairwise hidden representation preserves useful interface-specific metric information more effectively than the node-embedding-based MPNN representations once direct final-head access to raw edge attributes is removed.

### Explicit geometric cues reduce sample complexity:

**Figure 3.**
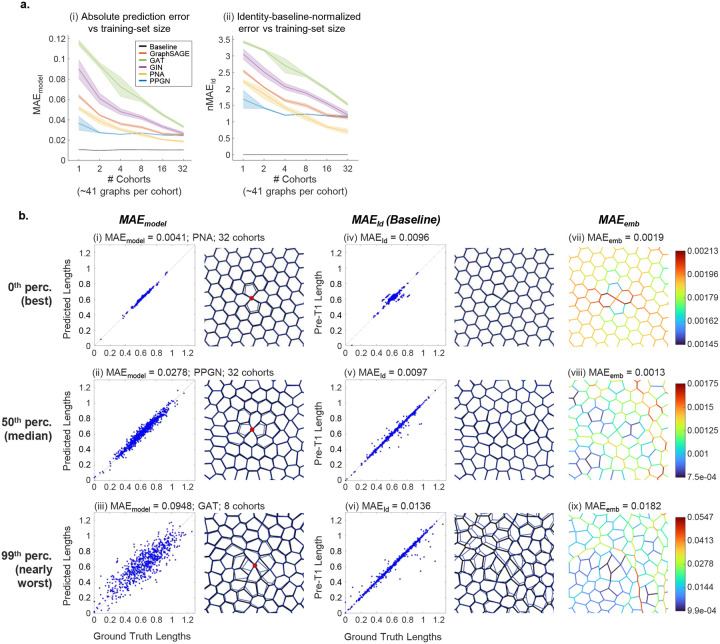
Edge-feature withholding reduces prediction accuracy and alters model ranking. **a.** Model performance versus training-set size when explicit edge features are withheld. Models were trained without pre-T1 edge lengths or the identity of the replaced T1 interface, leaving tissue topology and node features as inputs. (i) Mean graph-level absolute error for five GNN architectures trained on 1–32 cohorts, computed by first averaging edge-level absolute errors within each graph and then averaging graph-level $MAE_{\text{model}}$ values across test graphs and seeds. The identity baseline predicts post-T1 edge lengths as unchanged from pre-T1. (ii) Identity-baseline-normalized error, computed as graph-level ${log}_{2}$ ratios of ${MAE}_{\text{model}}(G)$ to ${MAE}_{\text{Id}}\left(G_{k}\right)$, followed by averaging across test graphs and seeds; values below zero indicate improvement over baseline. Curves show mean ± s.d. across n=5 random seeds. **b.** Representative best, median, and 99^th^-percentile test examples in the edge-feature-withheld regime, ranked by graph-level prediction error and displayed as in Fig. 1d.

In the fully specified vertex-model setting, relaxed post-T1 edge lengths depend on contact topology together with the geometric state and simulation setup, including the energy functional, mechanical parameters, boundary/domain geometry, and relaxation protocol. Pre-T1 edge lengths are therefore not uniquely required if the full geometric tissue state is otherwise specified, but they provide direct metric information that is absent from topology alone. We therefore asked how much of the relaxed post-T1 geometry could be statistically inferred when these direct metric cues were withheld from the GNN inputs. This topology-only condition is therefore a deliberately feature-withheld variant of the specified-perturbation task: the post-T1 contact graph encodes that a neighbor exchange has occurred, but the model is not given the pre-T1 metric geometry or an explicit marker of the remodeled interface. We retrained all models using the post-T1 contact topology but without pre-T1 edge lengths or an explicit label identifying the newly formed interface, forcing prediction of relaxed post-T1 lengths from connectivity and node features alone. Although this identity baseline uses pre-T1 edge lengths that are withheld from topology-only model inputs, it is used only as an evaluative reference for the magnitude of post-T1 edge-length changes, not as an admissible topology-only predictor. To keep the comparison matched to the changed input representation, we used a separate hyperparameter optimization for the edge-feature-withheld input regime, analogous to the optimization performed for the edge-featured regime (Methods; Supplementary Table S1). To calibrate these scales, Fig. 3b shows representative scatter plots and 2D embeddings from the 99th-percentile (nearly worst), median, and best test graphs. In the absence of explicit edge-length features, prediction accuracy was substantially lower: even with the largest dataset, none of the five architectures was able to reach its performance with the smallest dataset size when pre-T1 lengths were available. At the largest dataset, all models still remained above the identity baseline in average $nMAE_{Id}$, with PNA closest (0.70), followed by GraphSAGE (1.13), PPGN (1.16), GIN (1.21), and GAT (1.52). Notably, we also did not observe identity-fallback behavior in this condition; without access to pre-T1 edge lengths, the models could not rely on the copy-to-input strategy seen in the edge-feature task. Removing these cues also made the leaderboard data-dependent: PPGN performed best in the 4-cohort condition, whereas by 16 cohorts PNA had a clear advantage and remained best in the 32-cohort condition, with GraphSAGE and PPGN nearly tied behind it. Thus, in the current data, topology alone did not support sample-efficient learning of relaxed edge lengths; within the tested range, additional topology-only data did not recover the performance obtained from only 41 edge-feature training graphs.

### Prediction performance depends on mechanics, tissue size, tissue order, and perturbation geometry:

**Figure 4.**
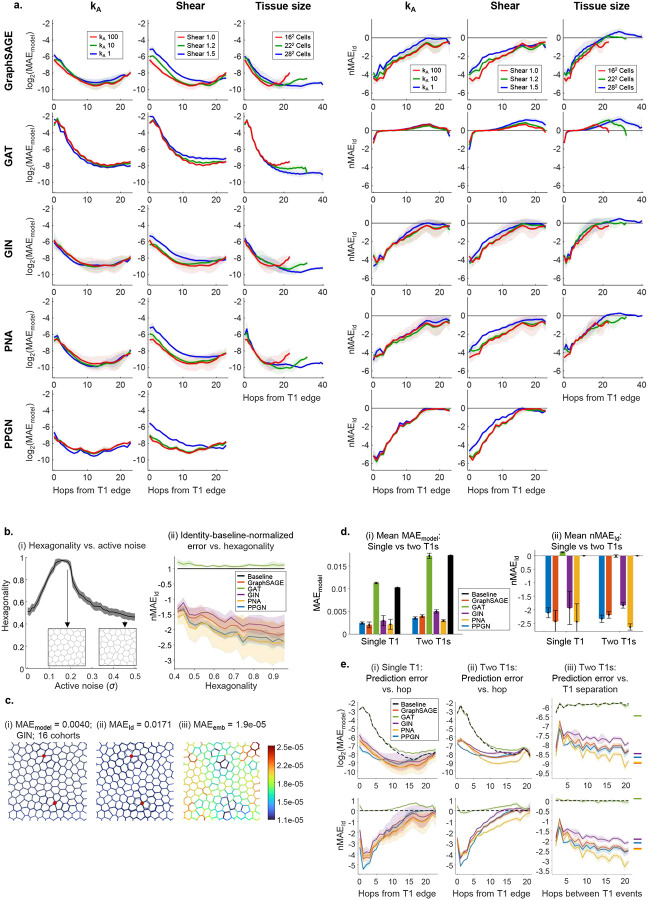
Tissue mechanics, geometry, order, and perturbation complexity shape prediction difficulty. **a.** Hop-resolved prediction error across controlled regime changes. Rows show GNN architectures; columns show area constraint coefficient ($k_{A}=100,10,1$), imposed shear (1.0, 1.2, 1.5), and tissue size (16^2^ = 256, 22^2^ = 484, 28^2^ = 784 cells). Errors are plotted versus edge-hop distance from the T1. Left panels show log-transformed raw error, computed by applying ${log}_{2}$ after graph-level $MAE_{\text{model},s}(h;G)$ values were averaged across test graphs within each seed, followed by averaging across seeds. Right panels show identity-baseline-normalized error, computed as graph-level ${log}_{2}$ ratios of $MAE_{\text{model},s}(h;G)$ to the hop-specific identity denominator $MAE_{Id}\left(h;G_{k}\right)$, followed by averaging across test graphs and seeds. Curves show mean ± s.d. across n=5 seeds. PPGN was not evaluated for larger tissue sizes because of computational cost. **b.** Tissue order analysis. (i) Hexagonality, the fraction of six-neighbor cells, versus active-noise parameter σ, with representative ordered and disordered tissues at σ=0.18 and σ=0.45. (ii) Identity-baseline-normalized error versus hexagonality, computed as graph-level ${log}_{2}$ ratios of $MAE_{\text{model}}(G)$ to the condition-specific identity denominator, followed by averaging across test graphs and seeds, using 40 test graphs from a balanced 400-graph dataset. **c.** Representative two-T1 prediction: the median-error GIN prediction from the 16-cohort benchmark. Panels show (i) embedded prediction overlaid on ground truth, (ii) identity-baseline geometry overlaid on ground truth, and (iii) embedding residuals. Color conventions follow Fig. 1d. **d.** Whole-graph prediction error for single- and two-T1 benchmarks using models trained on 16 cohorts: (i) raw $MAE_{\text{model}}$, averaged arithmetically across test graphs and seeds, and (ii) identity-normalized error, computed as graph-level ${log}_{2}$ ratios followed by averaging across test graphs and seeds. Bars show mean ± s.d. across n=5 seeds. **e.** Spatial error distribution for (i) single-T1 tissues, (ii) two-T1 tissues measured from the nearest T1, and (iii) two-T1 error versus the edge-hop separation between T1 events. Top panels show log-transformed raw error; bottom panels show identity-normalized error computed from graph-level ${log}_{2}$ ratios. Dashed black curves indicate the identity baseline; short horizontal marks in e(iii) show mean single-T1 performance.

We next asked whether model performance depends on measurable or controlled properties of the tissue and perturbation context. We first tested whether the benchmark conclusions were specific to the original vertex-model setting by varying the area-elasticity coefficient $k_{A}$, global shear, and tissue size (Fig. 4a). These analyses were not intended as exhaustive parameter sweeps; instead, they ask whether the spatial error structure and architecture-dependent behavior persist when the input-output mapping changes. For each condition, models were retrained with the previously optimized hyperparameters, so these are not out-of-distribution tests of models trained only on the original dataset.

Changing $k_{A}$, which controls how strongly cell areas are constrained to remain near the preferred area, altered both absolute and identity-normalized error. Lowering $k_{A}$ changed the mechanical response to a T1 and also changed the identity baseline itself, so raw MAE and normalized performance did not always move together. PPGN was comparatively robust across the tested $k_{A}$ values, whereas PNA, GIN, and particularly GraphSAGE lost normalized performance as $k_{A}$ decreased. GAT remained near or above the identity baseline throughout. Thus, architecture ranking should not be treated as a universal property of the model class, but as conditional on the mechanical regime.

Global area-preserving shear also changed prediction difficulty. Increasing shear generally increased raw error and shifted the hop-resolved profiles, but most non-GAT models still remained below the identity baseline at the graph level. At the strongest shear, GAT was worse than baseline, whereas PPGN, GraphSAGE, GIN, and PNA still retained better-than-baseline normalized performance. The hop-resolved curves are important here, because graph-level averages can hide changes in where each model approaches the identity baseline.

Tissue size produced a particularly important effect. Larger tissues extended the available hop-distance range, and near-T1 edges became a much smaller fraction of the graph, even though the absolute number of near-hop edges was similar across sizes. Consequently, graph-level averages increasingly reflected far-field behavior. PPGN was excluded from this analysis because of computational cost at larger graph sizes. Among the remaining models, GAT improved in raw MAE as tissue size increased but remained near or above baseline after normalization, while GraphSAGE, GIN, and PNA stayed below baseline but showed weaker normalized performance in larger tissues. Thus, tissue size should be interpreted as changing the spatial extent and weighting of the prediction problem, not merely as adding more locally equivalent examples.

We next examined tissue order, quantified by hexagonality, the fraction of cells with six neighbors. In our simulations, hexagonality varied from highly disordered tissues to nearly hexagonal packings, with a unimodal dependence on active noise and maximal order near intermediate noise levels (Fig. 4b(i)). We therefore trained and evaluated models on a balanced hexagonality dataset and asked whether $nMAE_{Id}$ depended on tissue order.

Across models, prediction accuracy improved as hexagonality increased (Fig. 4b(ii)). This trend indicates that ordered tissues, with more regular local neighborhoods and mechanical environments, are easier to predict after a T1 transition. The effect was architecture-dependent: PNA and PPGN generally achieved the strongest normalized performance, GraphSAGE and GIN were intermediate, and GAT remained close to or above the identity baseline over most hexagonality bins. Thus, hexagonality is useful as a post hoc descriptor of expected prediction difficulty, even though in real tissues it is usually a property of the biological system rather than a tunable experimental variable.

Finally, we asked how prediction changes when the perturbation contains two simultaneous T1 transitions rather than one; this perturbation geometry is demonstrated in Fig. 4c, and the resulting performance changes are quantified in Fig. 4d,e. Raw MAE increased for most models in the two-T1 setting, but the identity baseline worsened substantially as well (Fig. 4d(i)). As a result, normalized performance improved for some models, especially PNA and PPGN, while GraphSAGE and GIN remained better than baseline but with weaker normalized performance than in the single-T1 benchmark (Fig. 4d(ii)). When distance was measured from the nearest T1, the overall near-to-far structure was preserved, but the detailed profiles changed by architecture (Fig. 4e(i–ii)). Stratifying by the distance between the two T1 events showed that difficulty also depends on perturbation geometry (Fig. 4e(iii)), with the strongest errors occurring when the two events are close but not immediately adjacent. We interpret this conservatively as evidence that multiple perturbation sites alter the spatial distribution of learnable signal in an architecture-specific way.

#### Embedding reconstruction error is empirically bounded by edge-length prediction error:

Because the models predict edge lengths independently, a set of predicted lengths is not guaranteed to correspond exactly to a realizable two-dimensional tissue geometry. We therefore asked, within the initial edge-featured benchmark dataset, how graph-level edge-length prediction error relates to the downstream error introduced when predicted edge lengths are embedded into a two-dimensional tissue layout. For each test-graph prediction, we compared two quantities: the graph-level prediction error, $MAE_{\text{model}}$, and the embedding reconstruction error, $MAE_{emb}$, defined as the mean absolute difference between the edge lengths in the optimized 2D embedding and the model-predicted edge lengths that the embedding procedure was trying to realize.

**Figure 5.**
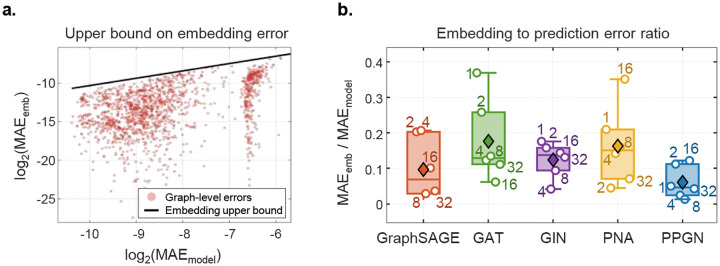
Embedding residuals are empirically bounded by edge-length prediction error. **a.** Relationship between graph-level prediction error ($MAE_{\text{model}}$) and embedding error ($MAE_{\text{emb}}$) in the reference edge-featured benchmark. Each point represents one test-graph prediction. Both axes are shown on a log scale. The solid line shows the empirical 100%-coverage upper envelope: ${\text{log}}_{2}\left(MAE_{emb}\right)\leq -0.7792+0.9530\cdot {\text{log}}_{2}\left(MAE_{\text{model}}\right)$, or equivalently $MAE_{emb}\leq 0.5827\cdot MAE_{\text{model}}^{0.9530}$. **b.** Embedding-error ratio, $MAE_{\text{emb}}/MAE_{\text{model}}$, by architecture and cohort size. Boxes summarize six cohort-level mean ratios per model, corresponding to 1, 2, 4, 8, 16, and 32 training cohorts. Box boundaries indicate the 25^th^ and 75^th^ percentiles, center lines indicate medians, whiskers extend to the most extreme values within 1.5× the interquartile range, open circles show individual cohort-level means labeled by cohort count, and filled diamonds show cohort-balanced means.

Across all model predictions and training-set sizes in the reference edge-featured benchmark, the maximal observed embedding error increased systematically with graph-level prediction error (Fig. 5a). In log-log space, all evaluated predictions fell below the following empirical coverage envelope:

(7) $${log}_{2}\left(MAE_{emb}\right)\leq -0.7792+0.9530\cdot {log}_{2}\left(MAE_{\text{model}}\right)$$

Or equivalently:

(8) $$MAE_{emb}\leq 0.5827\cdot MAE_{\text{model}}^{0.9530}$$

Thus, within the evaluated predictions, larger edge-length prediction errors were associated with larger possible embedding residuals. The embedding procedure did not introduce large additional errors beyond this empirical envelope. However, models with similar graph-level prediction error did not necessarily produce predicted edge-length fields that were equally easy to realize geometrically. We therefore quantified the embedding reconstruction ratio:

(9) $$R=\frac{MAE_{\text{emb}}}{MAE_{\text{model}}},$$

where the numerator measures the mismatch between the optimized 2D embedding and the model-predicted edge lengths, and the denominator measures the mismatch between the model-predicted and ground-truth post-T1 edge lengths. Thus, lower values indicate that, per unit prediction error, the predicted edge-length field is more compatible with a consistent two-dimensional embedding. This revealed architecture-dependent geometric realization fidelity (Fig. 5b). The cohort-balanced mean ratio was lowest for PPGN (R=0.060), followed by GraphSAGE (R=0.096), GIN (R=0.124), PNA (R=0.163), and GAT (R=0.176). Median-across-cohort ratios gave a similar ordering, with PPGN again lowest (R=0.046) and GAT and PNA among the highest. Except for GAT, which showed a stronger decrease in (R) with training-set size, this ratio showed little monotonic dependence on cohort count, suggesting that realization fidelity is primarily architecture-dependent in this benchmark.

These results show that graph-level prediction accuracy and geometric realization fidelity are related but not redundant. ${MAE}_{\text{model}}$ measures edge-length prediction error, whereas $MAE_{\text{emb}}$ measures how much additional distortion is required to embed the predicted length field as a two-dimensional tissue geometry. Thus, two models can have similar edge-length accuracy but differ in whether their residual errors are geometrically compatible or embedding-sensitive. Throughout the manuscript, embedded tissues are therefore used as post hoc visualizations, while all primary quantitative comparisons remain based on direct edge-length predictions before embedding. Together, these analyses extend the benchmark from accuracy alone to a geometric consistency check on the predicted tissue response.

## DISCUSSION

This study shows that GNN performance in tissue-remodeling prediction is governed not only by architecture, but also by feature availability, tissue structure, perturbation geometry, and the spatial distribution of the response. Using a controlled vertex-model benchmark, we evaluated how different GNN architectures learn the tissue-wide relaxation map produced by a prescribed T1 transition. The benchmark isolates a conditional-response problem: given a graph-structured representation of a tissue and local perturbation, predict the relaxed post-event length of every cell-cell interface. In the edge-featured version of this task, the flipped-edge label serves as a controlled conditioning cue for the specified perturbation, not as an oracle for natural event discovery. This controlled setting reveals not just which models perform well, but why performance changes across data regimes, input representations, and tissue conditions.

The benchmark does not support a single architecture ranking independent of context. With explicit edge-level geometry available, PNA and PPGN were most sample-efficient in the lowest-data regime, whereas GraphSAGE and GIN improved with additional training data and reached competitive performance in larger datasets. In the topology-only setting, where models received post-T1 connectivity without explicit edge-length features, performance dropped sharply and the architecture ranking changed. These results argue that model choice for tissue prediction should be treated as conditional on the available features, dataset size, and tissue regime rather than as an intrinsic property of the architecture alone.

The topology-only analysis provides one of the clearest practical lessons of the benchmark. In this setting, models received the post-T1 contact topology but not explicit pre-T1 edge lengths or a label marking the newly formed interface. In the fully specified simulation setting, the relaxed post-T1 geometry is obtained by applying the vertex-model relaxation protocol to a specified geometric tissue state. However, when explicit pre-T1 edge lengths and the T1-interface label were withheld from the GNN inputs, the models had to infer relaxed edge lengths from connectivity and node-level structural features alone. Even with the largest dataset tested, this feature-withheld setting did not recover the accuracy achieved by edge-featured models trained on the smallest dataset. This result distinguishes theoretical computability by a mechanistic model from practical learnability by finite-data GNNs. Explicit edge-level geometry did not merely improve performance by a small amount; it changed sample efficiency, model ranking, and failure mode.

The weak performance of GAT should therefore be interpreted as a property of the implemented attention-based baseline, not as evidence that attention mechanisms are intrinsically unsuitable for tissue prediction. More generally, the benchmark cautions against explaining model behavior from architecture names alone: performance can depend strongly on how geometric features are incorporated into message-passing, normalization, and edge-level readout.

Spatially resolved, baseline-relative evaluation exposed a failure mode that graph-level mean error would miss. In the length-informed task, several models switched from predicting post-T1 relaxation near the perturbation to copying supplied pre-T1 edge lengths in distal regions. The counterfactual perturbation experiment confirmed that this was literal input copying. The ablation analysis showed that the shortcut route is architecture-specific: in MPNNs, fallback depends strongly on direct final-head access to raw pre-T1 edge length, whereas in PPGN disabling the first raw-input skip was not sufficient to eliminate fallback. This suggests that PPGN can encode pre-T1 length into pairwise hidden channels and preserve it through later blocks. Thus, metric input pathways support useful near-event prediction but can also enable identity-like copying where post-T1 changes are weak.

Fallback is therefore not simply a generic loss of accuracy; it is a specific shortcut exposed by the benchmark. In distal regions where true pre-to-post changes are small, copying the pre-T1 edge length can be locally near-optimal under mean absolute error, but it limits the model’s ability to resolve weak long-range tissue responses. This matters when the goal is to compare perturbations, infer spatial response fields, or perform multi-step rollouts, where identity-biased predictions could accumulate or suppress subtle tissue-scale changes. The broader methodological lesson is that tissue-dynamics benchmarks should report spatially resolved and baseline-relative metrics, not only graph-averaged prediction error, and should test whether apparent accuracy depends on direct input-copying routes. At the same time, the ablations show that simply disrupting direct metric shortcuts is not a satisfactory solution: in MPNNs, removing final-head access to raw edge attributes degraded prediction, whereas in PPGN, disabling the first raw-input skip was insufficient to eliminate fallback. Future approaches should therefore aim to retain useful geometric input while discouraging trivial identity mappings, for example through residual-response targets, distance-aware losses, perturbation-focused weighting, or architectures that separate input-length encoding from direct length copying.

Prediction difficulty was also shaped by tissue structure. Hexagonality provided a simple descriptor of expected prediction error: ordered tissues, with more stereotyped local neighborhoods and mechanical environments, were easier to predict, whereas disordered tissues produced larger errors. In experimental applications, such structural descriptors could be used to stratify datasets, interpret model performance, and identify regimes in which additional examples, richer features, or different architectures may be needed.

The regime analyses extend this conclusion beyond tissue order. Changing area elasticity, imposed shear, tissue size, or the number and spacing of T1 events altered raw errors, identity-normalized errors, and hop-resolved profiles in different ways. This is why graph-level averages alone are insufficient: larger tissues, for example, increasingly weight far-field behavior, whereas multiple T1s redistribute the spatial structure of the response. These analyses support using the benchmark diagnostically, to ask which architectures remain accurate under the tissue mechanics, size, feature availability, and perturbation geometry relevant to a given biological application.

The benchmark also clarifies how prediction error should be interpreted when moving toward living tissues. Here, error measures how closely a model approximates a fully specified vertex-model input-output map. In experimental tissues, residual error could also reflect unmeasured biological state variables or measurement uncertainty, including contractility, adhesion, polarity, signaling, growth, external loading, segmentation or tracking error, imprecise event timing, incomplete relaxation, or unresolved concurrent rearrangements. These factors are not merely sources of noise: when measured, they can become informative node-, edge-, vertex-, temporal-, or graph-level features. The geometry-only benchmark should therefore be viewed as a controlled baseline for isolating graph-learning behavior, rather than as a complete representation of biological tissue state.

Future applications to live data can build on this benchmark in two ways. First, the input representation can be expanded beyond topology and geometry to include system-specific measurements such as local motion, protein localization, polarity markers, lineage information, cell-cycle state, or externally imposed mechanical conditions. These additions would allow feature ablations to test which measured variables improve prediction beyond geometric context alone. Second, biological interpretation will require validation beyond predictive accuracy. Model attributions, feature ablations, or activation maps should be treated as hypotheses about informative variables and tested against held-out embryos, mutants, mechanical or optogenetic perturbations, or independent measurements of force- or polarity-related quantities. This will be especially important for multi-step prediction, where small one-step edge-length errors may accumulate during sequential remodeling.

The benchmark also separates edge-level prediction from full geometric reconstruction. The models predict interface lengths edge by edge, and predicted length sets need not correspond exactly to a valid two-dimensional epithelial tiling. For this reason, all quantitative model comparisons are performed on predicted edge lengths before embedding; the embedding procedure is used only as a post hoc visualization and geometric-consistency diagnostic. Edge-level predictions can therefore be analyzed as learned response fields over the tissue graph, but they should not automatically be interpreted as complete physical tissue states. Future benchmark extensions could enforce geometric consistency more directly through differentiable embedding losses, penalties for non-realizable length sets, vertex-model energy regularization, or decoders that predict vertex coordinates or mechanically relaxed configurations rather than independent edge lengths.

Uncertainty quantification is an important extension of this benchmark. In the current study, each input graph has a deterministic target because the post-T1 state is obtained by energy minimization under a fixed simulation protocol. This allows model errors to be interpreted directly as deviations from a known vertex-model response. Live tissues introduce additional uncertainty from active fluctuations, unmeasured biological variables, annotation error, and concurrent events. Future models should therefore estimate not only the predicted tissue response, but also prediction confidence, regions where additional measurements may be needed, and cases where the input lies outside the training distribution.

In summary, this benchmark shows that GNN performance in tissue-remodeling prediction is not determined by architecture alone. Explicit geometric features strongly affect sample efficiency, tissue disorder is associated with prediction difficulty, spatially resolved metrics reveal hidden copy-to-input strategies, and changes in mechanics, tissue size, and perturbation geometry can alter model behavior. These results suggest several principles for future benchmarks and experimental applications: match architecture choice to the available features and biological question, evaluate predictions against simple identity baselines, report where in the tissue models improve over baseline, and treat high mean accuracy as incomplete unless shortcut behaviors and geometric consistency have been tested. By providing a controlled and reproducible vertex-model testbed, this study establishes a diagnostic framework for evaluating graph-learning approaches to multicellular tissue remodeling and for designing more biologically complete benchmarks.

## METHODS

### Vertex model simulation:

We first define a reference vertex-model simulation protocol, which serves as the baseline for the main benchmark and for the parameter-variation analyses described below. To prepare required sets of in silico tissue samples, we used a two-dimensional (2D) vertex model of confluent cell monolayers that describes tissues as planar tiling of polygonal cells^22,23^. In our model, equilibrium cell configurations minimize a potential energy that penalizes the total length of edges as well as deviations of actual cell areas $A_{j}$ from a preferred area $A_{0}$. In dimensionless form, where lengths and the energy are expressed in units of $\sqrt{A_{0}}$ and $\gamma_{0}\sqrt{A_{0}}$, respectively ($\gamma_{0}$ being the static interfacial line tension), the energy reads:

(10) $$w=\sum_{e\in \text{edges}}l_{e}+\sum_{j\in \text{cells}}k_{A}{\left(A_{j}-1\right)}^{2}.$$

Here, w is the dimensionless tissue mechanical energy, $l_{e}$ is the length of cell-cell interface $e,A_{j}$ is the area of cell $j,A_{0}$ is the preferred cell area, and $k_{A}$ is the dimensionless area-elasticity coefficient associated with cell-area compressibility; Unless otherwise stated, this reference protocol uses N=256 cells, periodic boundary conditions, $k_{A}=100$, no imposed tissue-scale shear beyond the affine adjustment needed to fit the initial hexagonal tiling into the square periodic domain, and one specified T1 perturbation followed by energy minimization. This model is well suited for our goal, since it describes cell mechanics in a fairly simplistic and non-specific manner. Indeed, it follows the conventional picture of tissue mechanics in which cellular cortical tension and cell-cell adhesion are both incorporated within a local effective interfacial line tension. The former contributes a positive tension, whereas the latter contributes a negative tension, indicating contractility of the cortex and the tendency to increase cell-cell contact due to adhesion, respectively.

Tissues in the reference dataset were simulated in a square periodic domain, starting from a regular hexagonal array of N=256 cells after the small affine adjustment required to fit the hexagonal tiling into the square periodic box. This initialization step is distinct from the imposed tissue-scale shear perturbations analyzed separately below. This highly correlated ordered sample is then made disordered and uncorrelated from the initial state by assuming fluctuations of interfacial line tensions. Biologically, these fluctuations mimic an active noise arising from stochastic molecular dynamics at the level of the edge actomyosin^24^. In particular, these fluctuations are modelled by active force dipoles whose magnitude $\Delta \gamma_{e}(t)$ undergoes persistent fluctuations as prescribed by the Ornstein-Uhlenbeck process^24,25,39^:

(11) $$\Delta {\dot{\gamma }}_{e}(t)=-k_{m}\Delta \gamma_{e}(t)+\xi_{e}(t),$$

where $\Delta \gamma_{e}(t)$ is the active fluctuation in interfacial tension along edge $e,k_{m}$ is the myosin turnover rate and $\xi_{e}(t)$ is the uncorrelated Gaussian white noise process associated with edge e, and sets the active-noise intensity, i.e., the amplitude of junctional tension fluctuations^24,25^. Due to active noise, the motion of cell vertices is no longer purely relaxational, since active noise allows the system to explore the energy landscape. In this equation, $r_{i}(t)$ is the position of vertex $i,{\dot{r}}_{i}(t)$ is its velocity, $-\nabla_{i}w$ is the conservative force derived from the dimensionless energy, and the second term is the active force contribution from fluctuating junctional tensions. The quantity $\nabla_{i}l_{e}$ denotes the gradient of interface length $l_{e}$ with respect to vertex position $r_{i}$:

(12) $${\dot{r}}_{i}(t)=-\nabla_{i}w-\sum_{e}\Delta \gamma_{e}(t)\nabla_{i}l_{e}.$$

Our simulations start at a high strength of active fluctuations, $\sigma_{\text{init}}=0.5$ (same for all simulations), which allows the system to frequently overcome local energy barriers to rearrange cells, thereby inducing a large number of T1 transitions, making the tissue highly disordered. The initial period of high activity, which can be referred to as the “melting” stage, is then followed by an instantaneous quench to a lower value of σ. Simulations then continue at this lower σ value until the system is sufficiently “thermalized”, meaning that the time-averaged statistical properties of the tissues (e.g., distribution of edge lengths and cell polygonality) are static. This “thermalization” step is followed by quenching the system instantaneously to $\sigma_{fin}=0$ and continuing the simulations without active noise. During this final step, tissues descend the potential-energy landscape and continue undergoing (passive) cell rearrangements by performing T1s on vanishingly short edges until, eventually, the system gets trapped in a local energy minimum with no more vanishingly short edges to undergo T1s. This protocol is intentionally idealized: it does not model the biological initiation of T1 transitions by polarity cues, active contractility, external loading, growth, proliferation, or signaling. Instead, it isolates the mechanical and geometric consequence of a specified neighbor exchange in a confluent tissue. The active-noise quench is used to generate diverse pre-T1 tissue states, whereas the benchmark T1 is imposed as a specified topological perturbation followed by energy minimization. The resulting task is therefore an equilibrium-to-equilibrium regression benchmark for testing how GNN architectures learn post-perturbation geometric changes across multiple cells.

We repeat this procedure for different values of σ, which provides us with a large variety of energy-minimized states with various degrees of disorder. For instance, the most disordered samples are obtained in the σ=0 and σ=0.5 cases. Indeed, at σ=0, tissues are provided with no activity during the “thermalization” step to transition from the initially melted state, prepared during the “melting” stage, to more ordered states, whereas at σ=0.5, tissues remain highly active throughout the first two simulation steps, which keeps them in fluidized states with frequent T1 transitions that maintain a high level of disorder. In contrast, samples with σ≈0.162, close to the solid-fluid transition^25^, are provided with just the right level of active noise during the thermalization step so as to undergo ordering from the initial melted sample. T1 transitions in these vertex-model simulations were implemented through a standard edge-threshold neighbor-exchange rule rather than through a cell-centered Voronoi or power-diagram formulation: when an interface length fell below a prescribed threshold, a neighbor exchange was triggered^22,23^. For the imposed benchmark perturbation, after preparing each energy-minimized pre-event tissue, one interface was selected uniformly at random, removed, and replaced by a new interface connecting the two formerly non-adjacent neighboring cells. The tissue was then relaxed by potential-energy minimization to a new local minimum, and the resulting post-T1 edge lengths were used as supervised-learning targets. The two-T1 analysis used the same relaxation procedure after imposing two specified neighbor exchanges.

### Dataset:

We generated a total of 32 cohorts of simulations, each consisting of 51 tissues sampled across edge noise levels (σ) ranging from 0 to 0.50 in increments of 0.01, yielding 1,632 tissue graphs in total. Each graph contained 256 cells, 512 vertices, and 768 edges. To test how prediction performance scales with training-set size, we constructed six predefined dataset-size conditions containing 1, 2, 4, 8, 16, or 32 simulation cohorts, respectively (Table 2). The 32-cohort condition used the full dataset. For the reduced-size conditions, the cohorts were assigned to non-overlapping subsets, so that each graph appeared in only one reduced-size condition and also in the full 32-cohort condition. Within each dataset-size condition, we designated a fixed subset of active-noise levels for validation (10%: σ=0.04,0.13,0.22,0.31,0.40) and testing (10%: σ=0.08,0.17,0.26,0.35,0.44), while using the remaining simulations for training (80%). This partitioning ensured that validation and test sets covered nearly the entire active-noise spectrum while limiting reuse of individual graphs across training-size comparisons. During data preparation, we identified 17 pairs of isomorphic graphs, defined as structurally identical cell-contact graphs arising from different initial seeds, and removed one graph from each pair, yielding a final dataset of 1,615 unique graphs. Lastly, each simulated tissue was encoded as a graph, where nodes represent cells, and edges represent cell–cell interfaces. Each graph was stored in tabular format, with one row per edge. For each edge, we recorded: (i) the indices of the two connected cells, (ii) the edge length before the T1, (iii) whether this was the replaced edge (Boolean flag), and (iv) the edge length after the T1. This representation preserves both the topology of the tissue and the dynamics of local edge changes, and is compatible with the input expectations of all GNN architectures we tested.

### GNN architectures and implementations:

We benchmarked five GNN architectures selected to span a range of expressive power, implementing each according to its peer-reviewed specification with minimal, architecture-specific adjustments to ensure stable optimization. The first four architectures—GraphSAGE^32^, GAT^31^, GIN^30^, and PNA^29^—are all MPNNs. The fifth architecture, PPGN^33^, departs from message-passing by operating on pairwise node–node tensors (N×N feature maps) with permutation-equivariant layers. This higher-order formulation is provably more expressive than standard MPNNs, enabling it to capture global connectivity motifs in tissues (e.g., multi-cell connectivity patterns) that are not recoverable from local aggregation alone, though at the cost of greater memory and runtime demands. A summary of the distinguishing properties of all five architectures is provided in Table 3.

We next standardized the implementation and readout choices needed to compare the five architectures on the same edge-length regression task. For the MPNNs, edge-level predictions were produced with a linear edge-regression head applied to the concatenation of the two endpoint embeddings and the corresponding edge attributes. This readout was trained directly on physical edge lengths, matching the continuous regression target of the benchmark. To make hop-resolved comparisons interpretable across MPNN architectures, we fixed the number of message-passing layers to 16 for all MPNNs. Because message-passing depth directly constrains the maximum topological distance over which information can propagate, fixing depth prevents differences in effective receptive field from reflecting architecture-specific hyperparameter choices rather than architectural inductive bias.

PPGN was treated separately because it does not follow the same node-embedding/message-passing/readout structure as the MPNNs. Instead, PPGN operates on pairwise node-node tensors and applies permutation-equivariant blocks with internal skip connections. We therefore used the published PPGN architecture without adding the MPNN edge-regression head, Jumping Knowledge readout, or per-graph instance normalization. Preliminary validation experiments indicated that applying the same per-graph instance-normalization strategy used for the MPNNs reduced PPGN performance in this task, so PPGN was trained with dataset-level normalization, L1 loss on the full output mask, and gradient clipping to prevent exploding-gradient events.

After defining these architecture-specific choices, we standardized the graph representation as much as possible across models. For the MPNN models, each node, corresponding to a cell, was initialized with a Local Degree Profile consisting of the node degree and the minimum, maximum, mean, and standard deviation of neighboring node degrees^40^. We also supplied Laplacian positional encodings derived from the lowest 30 non-trivial eigenvectors of the normalized graph Laplacian, providing a structural proxy for position within the tissue graph^41^. Because the sign of each Laplacian eigenvector is arbitrary, random sign choices would make otherwise identical graph representations differ across preprocessing or prediction runs. We therefore fixed the sign of each eigenvector using a canonical convention, making positional encodings deterministic and preventing arbitrary sign flips from contributing to model differences.

For the MPNNs, we used per-graph instance normalization^42^ and Jumping Knowledge concatenation^43^ to improve convergence and information propagation across the fixed 16-layer architectures. For the MPNN models, dropout^44^ was treated as an optimized hyperparameter, whereas PPGN did not use dropout; generalization was controlled through validation-based model selection, early stopping, repeated random seeds, and fixed train/validation/test splits. Target and degree-normalization quantities used during preprocessing were computed from the training split and stored with the trained model, so that validation and test graphs were transformed using training-set statistics rather than information from the full dataset.

### Hyperparameter optimization:

Hyperparameters were optimized using MATLAB *bayesopt* on the original 16-cohort reference dataset, separately for each architecture and separately for the two input regimes: the edge-featured regime, in which pre-T1 edge length and the T1-interface flag were provided, and the feature-withheld/topology-only regime, in which both of these edge features were withheld. This yielded 10 optimization runs. Hyperparameter selection used the predefined training and validation splits only; test sets were not used during optimization. The optimized hyperparameters were then frozen and reused for the corresponding downstream analyses. For all models, Bayesian optimization used 20 objective evaluations with 6 initial seed points per model/input condition. Each trial used training seed 0, a 120-epoch cap, early-stopping patience of 40 epochs, ReduceLROnPlateau patience of 20 epochs, and early-stopping min_delta = 1e-4.

For the MPNN models, optimized parameters were hidden-channel width in {64, 128}, dropout in {0, 0.1, 0.2}, batch size in {1, 2, 4}, and learning rate sampled log-uniformly over [1e-4, 1e-2]. Fixed settings included scheduler factor = 0.75, weight decay = 0, network depth = 16 layers, and validation MAE, equivalent to validation L1 error, as the optimization objective. MPNN models used internal per-graph instance normalization. For PPGN, optimized parameters were learning rate sampled log-uniformly over [1e-5, 1e-2], batch size in {2, 4, 8, 16, 32}, scheduler factor in {0.1, 0.2, …, 0.8}, and gradient clipping in {0.001, 0.01, 0.1, 1}. The PPGN architecture was fixed at block_features = [400, 400, 400] and depth_of_mlp = 2. Input pre-T1 edge lengths and output post-T1 edge lengths were z-scored when present. PPGN did not use internal per-graph instance normalization, as preliminary validation experiments showed that it reduced performance. All final reported models were retrained from scratch with the selected hyperparameters for five independent random seeds (0–4), with a 2000-epoch cap. The selected configurations are reported in Supplementary Table S1.

### Additional vertex-model regime datasets:

To test whether model behavior depended on the specific reference simulation regime, we generated additional datasets that varied one controlled aspect of the vertex-model setup or perturbation protocol at a time. These analyses used the same graph construction, train/validation/test split logic, edge-featured input representation, and supervised target definition as the reference benchmark. Unless otherwise stated, each condition was matched to the 16-cohort reference setting with pre-T1 edge lengths included, and models were retrained from scratch with five independent random seeds using the hyperparameters selected for the corresponding pre-T1 edge-length-included benchmark (Supplementary Table S1). Thus, these analyses test how fixed model configurations behave across altered tissue regimes, rather than introducing a separate hyperparameter-optimization problem for each condition.

For the mechanical-regime analysis, we varied the area-elasticity coefficient $k_{A}$, which controls the energetic penalty for deviations from the preferred cell area. The reference condition used $k_{A}=100$, taken from the original 16-cohort reference dataset with pre-T1 edge lengths included, and we generated matched de novo datasets with $k_{A}=10$ and $k_{A}=1$. All other simulation and graph-construction settings were kept the same as in the 16-cohort reference condition with pre-T1 edge lengths included. For the anisotropic-deformation analysis, we compared the undeformed reference condition λ=1 with two area-preserving rectangular box deformations λ=1.2 and λ=1.5. Starting from relaxed $N=256,k_{A}=100$ reference tissues, we rescaled the periodic domain and vertex coordinates as $L_{x}\to \lambda L_{x}$ and $L_{y}\to L_{y}/\lambda$, preserving tissue area while changing the global aspect ratio. The deformed tissues were then relaxed with active noise turned off before the benchmark T1 perturbation was imposed. Pre-T1 edge lengths were recorded after this post-deformation relaxation, so the model inputs corresponded to the relaxed anisotropically deformed tissue rather than to the original square-domain tissue. After imposing the specified T1 transition, each tissue was relaxed again to obtain the post-T1 target edge lengths.

For the tissue-size analysis, we compared the reference N=256 tissues with larger tissues containing N=484 and N=784 cells. These sizes correspond to square-cell-count initializations of 16^2^, 22^2^, and 28^2^ cells, respectively, preserving the square-domain construction used in the reference simulations. Matched de novo datasets were generated for the larger tissue sizes using the same simulation, splitting, graph-construction, and edge-feature representation logic as in the 16-cohort reference condition with pre-T1 edge lengths included. PPGN was excluded from the tissue-size analysis because its pairwise node-node tensor representation becomes substantially more expensive for larger graphs.

For the perturbation-complexity analysis, we generated a two-T1 dataset using the same simulation settings as the 16-cohort reference condition with pre-T1 edge lengths included, but imposed two randomly selected T1 transitions in each tissue instead of one before relaxation. The post-perturbation target was again defined as the relaxed edge length of every cell-cell interface after potential-energy minimization. For hop-resolved analyses in the two-T1 condition, distance was measured relative to the nearest imposed T1 interface, and performance was additionally stratified by the distance between the two imposed T1s.

For the tissue-order analysis, we constructed a balanced hexagonality subset from the original edge-featured single-T1 reference dataset. Hexagonality was defined as the fraction of cells with six neighbors. We selected 400 graphs to span the observed hexagonality range more evenly than the full reference dataset. Because this subset had different graph counts from the main dataset-size conditions, it used the same train/validation/test splitting logic but a separate split: 80% of graphs for training, 10% for validation, and 10% for testing. Models were retrained on this subset using the same graph representation, supervised target, and pre-T1 edge-length-included hyperparameters used for the corresponding reference analyses.

### 2D Embedding of predicted tissues:

Because our vertex-model simulations represent planar epithelial tissues, any physically plausible prediction of tissue geometry should admit a consistent 2D embedding. However, predicted edge lengths from a GNN do not necessarily define a realizable planar layout, especially in the presence of small inconsistencies or accumulated noise. To enable visualization and potential downstream geometric analysis, we developed an agnostic tissue embedding algorithm that builds on the same frameworks as our vertex model (Methods) to find a planar embedding of the predicted tissue state with edge lengths as close as possible to the predicted values. Using gradient descent, we simulate vertex movements to minimize a penalty function:

(13) $$P=\sum_{e\in \text{edges}}{\left(l_{e}-L_{e}\right)}^{2}.$$

This function penalizes deviations of edge lengths $l_{e}$ of the embedded tissue from the predicted values $L_{e}$. The resulting embedding can be viewed as the 2D realization of the tissue that is closest to the predicted tissue in terms of sum of squared differences between embedded and predicted edge lengths. As the initial configuration of the tissue in the optimization of the term, we use the pre-T1 simulated geometry after locally replacing the T1 site with the post-T1 configuration. If a tissue with predicted edge lengths is embeddable in 2D, the penalty function equals the minimal possible value (0). The embedding is applied uniformly for visualization purposes only, and all model evaluations are based on edge-length predictions prior to embedding.

#### Performance metrics and identity baselines:

For each tissue graph G and random seed s, we evaluate predictions of post-T1 edge lengths using the mean absolute error:

(14) $${MAE}_{\text{model},s}(G)=\frac{1}{\left|E_{G}\right|}\sum_{e\in E_{G}}\left|{\overset{ˆ}{l}}_{e,S}-l_{e}^{\text{post}}\right|.$$

For each graph G, the identity-baseline error was defined by predicting each post-T1 edge length as its matched pre-T1 length:

(15) $${MAE}_{Id}(G)=\frac{1}{\left|E_{G}\right|}\sum_{e\in E_{G}}\left|l_{e}^{pre}-l_{e}^{\text{post}}\right|.$$

For each dataset-size condition k and random seed s, raw model error was averaged arithmetically over the corresponding test set $G_{\text{test}}^{\left(k\right)}$:

(16) $${\bar{MAE}}_{\text{model},s}^{\left(k\right)}=\frac{1}{\left|G_{\text{test}}^{\left(k\right)}\right|}\sum_{G\in G_{\text{test}}^{\left(k\right)}}MAE_{\text{model},s}(G).$$

The identity denominator was computed as the equal-graph-weighted average identity error over the same test set:

(17) $${MAE}_{Id}\left(G_{\text{test}}^{\left(k\right)}\right)=\frac{1}{\left|G_{\text{test}}^{\left(k\right)}\right|}\sum_{G\in G_{\text{test}}^{\left(k\right)}}MAE_{Id}(G).$$

For each test graph G, model, and random seed, identity-normalized error was computed as a graph-level log-ratio:

(18) $$nMAE_{Id,S}\left(G,G_{\text{test}}^{\left(k\right)}\right)={log}_{2}\left(\frac{MAE_{\text{model},s}(G)}{MAE_{Id}\left(G_{\text{test}}^{\left(k\right)}\right)}\right).$$

The plotted normalized value was then obtained by averaging these graph-level log-ratios over test graphs and random seeds:

(19) $$nMAE_{Id}^{\left(k\right)}=\frac{1}{5}\sum_{s=1}^{5}\frac{1}{\left|G_{\text{test}}^{\left(k\right)}\right|}\sum_{G\in G_{\text{test}}^{\left(k\right)}}nMAE_{Id,s}\left(G,G_{\text{test}}^{\left(k\right)}\right).$$

Thus, $nMAE_{Id}=0$ indicates parity with the identity baseline, $nMAE_{Id}<0$ indicates improvement over identity, and $nMAE_{Id}>0$ indicates worse performance than identity. Because the logarithm is applied before averaging over graphs, this value is a mean log-ratio, not the log ratio of the arithmetic mean model error to the arithmetic mean identity error.

For hop-resolved analyses (Fig. 2 and Supplementary Figs. S1–S2), hop distance is computed on the line graph L(G), the edge-to-edge adjacency of the tissue graph G. In L(G), two tissue edges are adjacent if they share a common cell, allowing distance to be measured by traversing connected cell-cell interfaces. Hop 0 corresponds to the T1 interface: the removed interface in the pre-T1 graph and the newly formed interface in the post-T1 graph. Let $E_{h}(G)\subseteq E_{G}$ denote the set of tissue edges at hop distance h from the T1 edge in G, and let $G_{k}(h)=\left\{G\in G_{k}:\left|E_{h}(G)\right|>0\right\}$, denote the set of graphs containing at least one edge at hop h. For each graph, hop-resolved model error and the corresponding identity-baseline error were first computed within graph:

(20) $$MAE_{\text{model},s}(h;G)=\frac{1}{\left|E_{h}(G)\right|}\sum_{e\in E_{h}(G)}\left|{\overset{ˆ}{l}}_{e,s}-l_{e}^{\text{post}}\right|,$$

(21) $$MAE_{Id}(h;G)=\frac{1}{\left|E_{h}(G)\right|}\sum_{e\in E_{h}(G)}\left|l_{e}^{pre}-l_{e}^{\text{post}}\right|.$$

For raw hop-resolved MAE curves, graph-level errors were then averaged arithmetically across graphs and finally across seeds:

(22) $${MAE}_{\text{model}}\left(h;G_{k}\right)=\frac{1}{5}\sum_{s=1}^{5}\frac{1}{\left|G_{k}(h)\right|}\sum_{G\in G_{k}(h)}{MAE}_{\text{model},s}(h;G).$$

For log-transformed raw-error curves, the logarithm was applied after graph averaging within each seed, and the resulting values were then averaged across seeds:

(23) $${log}_{2}\left(MAE_{\text{model}}\left(h;G_{k}\right)\right)=\frac{1}{5}\sum_{s=1}^{5}{\text{log}}_{2}\left(\frac{1}{\left|G_{k}(h)\right|}\sum_{G\in G_{k}(h)}MAE_{\text{model},s}(h;G)\right).$$

The hop-specific identity denominator was computed as the equal-graph-weighted identity error at that hop:

(24) $$MAE_{Id}\left(h;G_{k}\right)=\frac{1}{\left|G_{k}(h)\right|}\sum_{G\in G_{k}(h)}MAE_{Id}(h;G).$$

For each test graph, model, and random seed, hop-resolved identity-normalized error was then computed as a graph-level log-ratio:

(25) $$nMAE_{Id,S}\left(h;G,G_{k}\right)={log}_{2}\left(\frac{MAE_{\text{model},s}(h;G)}{MAE_{Id}\left(h;G_{k}\right)}\right).$$

The plotted hop-resolved $nMAE_{Id}$ value was obtained by averaging these graph-level log-ratios over graphs and seeds:

(26) $$nMAE_{Id}\left(h;G_{k}\right)=\frac{1}{5}\sum_{s=1}^{5}\frac{1}{\left|G_{k}(h)\right|}\sum_{G\in G_{k}(h)}nMAE_{Id,s}\left(h;G,G_{k}\right).$$

Here, ${\overset{ˆ}{l}}_{e,s}$ is the GNN-predicted post-T1 length of edge e for random seed $s,l_{e}^{pre}$ is its pre-T1 length, and $l_{e}^{\text{post}}$ is its ground-truth post-T1 length.

## Supplementary Material

Supplementary Files

This is a list of supplementary files associated with this preprint. Click to download.

- Krajncetal.RevisionSupplementary.pdf
- Table2.docx
- Table1.docx
- Table3.docx

## Data Availability

The data package needed to reproduce the manuscript analyses is available on Zenodo at https://doi.org/10.5281/zenodo.21286579. The archive includes the processed graph datasets used for training, validation, and testing, train/validation/test splits, consolidated model predictions, saved graph-embedding outputs, trained model checkpoints, analysis tables, generated figures, and manuscript-specific diagnostic outputs.

## Code availability:

Custom code used in this study, including code for vertex-model simulations, dataset generation and assembly, GNN training and evaluation, tissue embedding, post-prediction analyses, and figure generation, is publicly available at https://github.com/Stern-Lab-UM/GNN-Benchmark-Code/. The repository includes the MATLAB top-level publication pipeline, Python implementations for the GNN models, C/C++ source code for tissue simulation and spring embedding, installation and setup files, and documentation describing the run modes, workflow stages, software environment, dataset splits, model hyperparameters, random seeds, and analysis parameters used in the manuscript. No access restrictions apply.
